# Combination strategies for pandemic influenza response - a systematic review of mathematical modeling studies

**DOI:** 10.1186/1741-7015-7-76

**Published:** 2009-12-10

**Authors:** Vernon J Lee, David C Lye, Annelies Wilder-Smith

**Affiliations:** 1 Center for Health Services Research, National University of Singapore, Singapore; 2 Biodefence Center, Ministry of Defence, Singapore; 3 Department of Clinical Epidemiology, Tan Tock Seng Hospital, Singapore; 4 National Center for Epidemiology and Population Health, Australian National University, Australia; 5 Department of Infectious Diseases, Tan Tock Seng Hospital, Singapore; 6 Department of Epidemiology and Public Health, National University of Singapore, Singapore

## Abstract

**Background:**

Individual strategies in pandemic preparedness plans may not reduce the impact of an influenza pandemic.

**Methods:**

We searched modeling publications through PubMed and associated references from 1990 to 30 September 2009. Inclusion criteria were modeling papers quantifying the effectiveness of combination strategies, both pharmaceutical and non-pharmaceutical.

**Results:**

Nineteen modeling papers on combination strategies were selected. Four studies examined combination strategies on a global scale, 14 on single countries, and one on a small community. Stochastic individual-based modeling was used in nine studies, stochastic meta-population modeling in five, and deterministic compartmental modeling in another five. As part of combination strategies, vaccination was explored in eight studies, antiviral prophylaxis and/or treatment in 16, area or household quarantine in eight, case isolation in six, social distancing measures in 10 and air travel restriction in six studies. Two studies suggested a high probability of successful influenza epicenter containment with combination strategies under favorable conditions. During a pandemic, combination strategies delayed spread, reduced overall number of cases, and delayed and reduced peak attack rate more than individual strategies. Combination strategies remained effective at high reproductive numbers compared with single strategy. Global cooperative strategies, including redistribution of antiviral drugs, were effective in reducing the global impact and attack rates of pandemic influenza.

**Conclusion:**

Combination strategies increase the effectiveness of individual strategies. They include pharmaceutical (antiviral agents, antibiotics and vaccines) and non-pharmaceutical interventions (case isolation, quarantine, personal hygiene measures, social distancing and travel restriction). Local epidemiological and modeling studies are needed to validate efficacy and feasibility.

## Background

Many countries have developed pandemic preparedness plans in response to the threat from pandemic influenza [1], to attempt containment of the virus or to reduce the pandemic's impact. The influenza A (H1N1-2009) pandemic has underscored the importance of such plans, with the World Health Organization (WHO) calling for the activation of pandemic plans worldwide [2]. Although the WHO has made public guidelines for developing pandemic plans [3], the comprehensiveness and standards of pandemic plans differ widely across different countries and continents [4-6]. To ensure the success of these plans, it is necessary to adopt a combination of different strategies.

Although there are existing historical data on the possible success of strategies used in previous pandemics such as personal hygiene, school and workplace closures, and social distancing, these are often anecdotal and difficult to interpret [7,8]. Mathematical models provide a platform for the assessment of multiple interventions in an environment where individual parameters can be altered. The recent increase in mathematical modeling studies on pandemic interventions suggests the effectiveness of these strategies and provides guidance for policy makers. Although the 2009 pandemic has spread rapidly, these combination strategies can be applied in populations yet to be severely affected, for the second wave, or for the next pandemic [9,10]. This systematic review aims to determine the individual components that constitute combination strategies, and the quantitative impact of these combination strategies in reducing pandemic spread and morbidity.

## Methods

This study explored available mathematical modeling publications on the effectiveness of combination strategies for an influenza pandemic. To obtain papers on the effectiveness of combination strategies, data for this review were identified by the authors through searches of the PubMed search engine for English language articles and articles translated into the English language. The authors used the following search terms to focus on modeling studies, and those which had a focus on pandemic preparedness and strategies - *influenza *and *pandemic *and (*preparedness *or strateg* or model*); *influenza *and *modeling *or *modelling*. The search included all published articles listed on PubMed from 1990 to 30 September 2009 - there were few articles on influenza pandemic planning or modeling before this period.

Abstracts were reviewed where available to determine if a study met the inclusion criteria and the full manuscript was obtained for further scrutiny. Snowball searches by hand were performed on the reference lists of articles meeting the inclusion criteria to find additional studies.

The inclusion criteria were primary mathematical modeling papers that compared and reported the quantitative effectiveness of combination strategies (two or more strategies used together) versus individual strategies for human pandemic influenza. Mathematical modeling papers were those which used quantitative predictive methods to determine the likely impact of strategies, and had descriptions of these methods which could be reproduced or verified. All influenza preparedness strategies were considered, including pharmaceutical and non-pharmaceutical public health strategies. These articles would allow clear comparison on the advantages of combination strategies over and above the impact of individual strategies. An explanation of some of the key strategies are found in the appendix.

Mathematical modeling articles that described the effectiveness of multiple singular strategies but did not analyze the quantitative effect of combination strategies were excluded. Articles that referred to general pandemic preparedness without quantitative evidence, or provided only qualitative discussion were also excluded. Reviews without primary data, articles in abstracts without full publication, and unpublished studies were excluded as their methodology and results could not be verified.

Mathematical models are based on input variables which are assumptions made based on available evidence in specific scenarios. One important assumption is the reproductive number (Ro), which is the average number of secondary infections generated by a single case in a completely susceptible population. No attempt was made in this review to homogenize data across studies for comparison; on the contrary, the heterogeneity of data provides public health professionals with evidence of the effectiveness of strategies across a wide range of assumptions and scenarios. We have instead listed the different types of models used, and the scenarios, interventions, and countries where they were applied.

## Results

**Figure 1:**
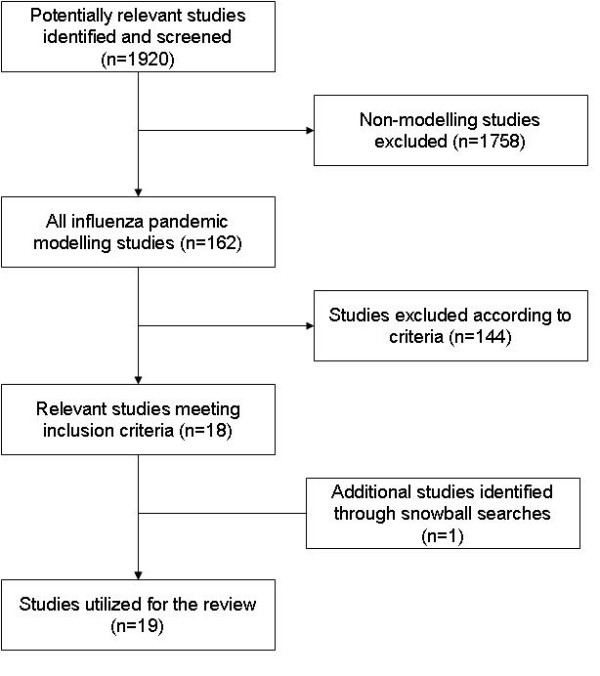
**Flow diagram for selection of combination strategy modeling studies**.

The search yielded a total of 1,920 papers including overlaps. Of these, 162 used mathematical modeling techniques and on closer review, 144 were excluded based on the exclusion criteria listed in Methods. The remaining 18 studies were included for analysis, together with one additional study identified from the snowball searches (Figure 1). The selected modeling papers that show the effectiveness of combination strategies in increasing the impact of individual strategies are listed in Additional files 1 and 2[11]. The following sections highlight key findings on the effectiveness of combination strategies in these modeling studies on pandemic influenza.

### Source containment

Zoonotic influenza such as H5N1 influenza is endemic in several countries, and there is interest in containing a highly virulent pandemic at the earliest sign of localized efficient human-to-human transmission. Two key modeling studies suggested a high probability of success for rapid containment of an influenza epicenter with combination strategies under favorable conditions [9,10]. These studies formed the basis for the epicenter containment strategies recommended by the WHO. Longini showed that antiviral prophylaxis alone could contain a pandemic influenza virus with reproductive number (Ro) less than 1.7; while 70% household quarantine alone was effective up to Ro of 1.7. A combination of quarantine and anti-viral prophylaxis was effective up to Ro of 2.1; while a combination of pre-pandemic vaccination, household quarantine and antiviral prophylaxis was effective for Ro of 2.4 [9]. Ferguson found that antiviral prophylaxis for contacts only would have a 90% chance of containing a virus with a Ro less than 1.25, while antiviral prophylaxis for contacts and all individuals in a 10 km zone would have a 90% chance with Ro less than 1.7 [10]. Combined anti-viral prophylaxis and either school and workplace closures or area quarantine provided a similar chance of containment with Ro of 1.7 to 1.8, while a combination of all three strategies would contain a virus with Ro of 1.9 and allow for greater initial surveillance errors [10].

### Reducing pandemic spread

Combination strategies can be used to reduce the global spread of the influenza virus [12,13]. Redistribution of limited antiviral drugs can help contain pandemics or reduce the global attack rate (AR) [12]. If global antiviral stockpiles are limited, non-cooperative strategies where countries keep their antiviral stockpiles for their own use can only contain a pandemic influenza virus with Ro less than 1.5; in contrast, if redistribution of 25% of stockpiles from countries that have them to countries that do not, a pandemic with Ro up to 1.9 may be contained, and overall AR reduced by 25% at higher Ro [12].

Another example of combination strategy is reduction of pandemic spread through air travel. Suspension of 99.9% of air travel can only delay individual national epidemics by up to four months, while a combination of local strategies reducing influenza transmission by 40% can delay pandemic spread by up to 10 months [13]. A combination of vaccination and travel restrictions may delay epidemic growth, allowing vaccination of susceptible individuals [14]. With a pandemic starting in July in Asia, the number of United States (US) metropolitan cases was 102.4 million - 0.1% daily vaccination alone reduced this to 73.0 million, and vaccination together with travel restriction reduced this to 56.9 million [14].

Combination strategies may have substantial impact in reducing the global spread of resistant viruses. For example, if the probability of emergence of anti-viral drug resistance was 1%, antiviral monotherapy was associated with overall AR of 67% and resistant AR (RAR) of 38% [15]. In contrast, early combination chemotherapy was associated with reduced AR of 58% and RAR of 2%, while sequential multi-drug chemotherapy was associated with AR of 57% and RAR of 3%.

### Mitigating pandemic impact

During the pandemic, several studies found that combination strategies delayed the spread of the virus, reduced the overall number of cases, and delayed and reduced the peak AR much more than individual strategies which may be ineffective if used alone [16-19].

A study using individual-based modelling in the United Kingdom and United States examined the effects of antiviral treatment and prophylaxis, vaccination, case isolation, household quarantine, school and workplace closure and travel restrictions in pandemics with Ro of 1.7 to 2.0. It found that external or internal travel restrictions alone would delay spread by two to three weeks only if more than 99% effective [16]. Reactive school and workplace closures alone did not impact on overall AR, but reduced peak AR by about 40%; antiviral treatment and prophylaxis within the household reduced overall AR by 35% and peak AR by 45%.; while household quarantine alone reduced overall AR by 10% and peak AR by 20%. Combination antiviral treatment and prophylaxis, and household quarantine reduced overall AR by 40% and peak AR by 60%. Combination school and workplace closure, antiviral treatment and prophylaxis, and household quarantine reduced overall AR by more than 60% and peak AR by more than 80%. Combination antiviral treatment and prophylaxis, school closure and 20% pre-pandemic vaccination reduced overall AR by more than 60% and peak AR by more than 75%. Combination antiviral treatment and prophylaxis, household quarantine, school and workplace closure, and effective border control reduced overall AR by more than 70% and peak AR by more than 90% [16].

Similarly, another individual-based stochastic simulation model in Chicago evaluating the effects of antiviral treatment and prophylaxis, quarantine, isolation, school closure, community and workplace social distancing showed that social distancing alone may reduce overall AR by 60% for pandemic Ro of 1.9 but combination antiviral treatment and prophylaxis, quarantine, social distancing, and school closure could reduce overall AR by more than 90% for similar pandemic Ro of 1.9 [17].

Another study in France examined the effects of antiviral treatment and household prophylaxis, vaccination, household quarantine, school and workplace closure at the individual and community level [20]. Treatment only with anti-viral drugs did not affect AR substantially. Antiviral prophylaxis of 90% of household contacts reduced AR by 50%. Vaccination of 70% of the population within one day reduced AR by 80%. A combination of antiviral treatment and prophylaxis, and household quarantine reduced AR by 90% [20].

An Australian individual-based stochastic simulation model assessed the effects of non-pharmacological pandemic mitigation measures of case isolation, school closure, workplace non-attendance and community contact reduction [21]. For a pandemic with Ro of two, school closures alone reduced AR by 20%, case isolation by 40%, workplace non-attendance by 15%, and social distancing by 25%. In contrast, combination of all these measures reduced AR by more than 95% [21].

A deterministic compartment model using *InfluSim *based on a small community of 100,000 population assessing the effects of antiviral treatment, case isolation and social distancing showed that case isolation and social distancing could reduce overall AR by 25%, and antiviral treatment alone by 20%, compared with a reduction of 40% with a combination of case isolation, social distancing and antiviral treatment [18]. The triple combination strategy could delay the peak by one month compared with 10 days for the first two strategies [18].

Another study using a deterministic model with a stochastic simulation component based on Italy examined the effects of household antiviral prophylaxis, pre-pandemic vaccination, and social distancing via closure of all schools, public offices and public meeting places [22]. In a pandemic with an attack rate of 35%, vaccination alone reduced AR by up to 10% even at vaccine efficacy levels of 70%; antiviral prophylaxis alone for even the entire pandemic duration reduced AR by up to 6% only; and social distancing alone reduced AR by less than 1%. However, a combination of all three measures reduced AR by up to 30% [22].

### Intervention effectiveness with changes in Ro

The relative success of interventions depends on the transmissibility of the pandemic, which is commonly reflected in the Ro. In an influenza pandemic with higher Ro, the effectiveness of interventions is reduced and individual interventions are commonly ineffective. However, across most scenarios, combination strategies maintain some effectiveness as shown clearly in the studies on containment by Longini [9] and Ferguson [10].

A stochastic agent-based discrete-time simulation model in the United States examining the effect of antiviral prophylaxis, vaccination, school closure and travel restriction found that for a pandemic influenza virus with Ro of 2.4, unlimited antiviral prophylaxis and best vaccination program may reduce cases by 64% and 34% respectively, while school closure within seven days of pandemic onset may reduce cases by 14%, social distancing within seven days by 6%, and travel restrictions exceeding 90% was ineffective [19]. However, a combination strategy of all of these measures may reduce cases by 99.8% [19]. The effectiveness of any strategy in delaying the pandemic or reducing the AR is highly dependent on the Ro. For example, for a pandemic with Ro of 1.6, individual strategies of prophylaxis, vaccination, or school closures had very high effectiveness [19]. However, once the Ro increased beyond 2.0 (which is similar to the Ro for the 1918 pandemic), individual strategies were much less effective, whereas combination strategies still maintained effectiveness across a range of Ro.

An individual-based model in Italy assessing the effects of household, school and workplace antiviral prophylaxis, vaccination, international air travel restriction, social distancing via school closure and closure of some public offices showed that without any interventions, importation of pandemic influenza would occur 37 to 77 days after the first case elsewhere in the world. Air travel restriction would delay introduction by one week to one month. For a pandemic with Ro of 1.7, travel restriction and social distancing did not affect overall AR, household prophylaxis reduced AR by 50%, and vaccination reduced AR by 0 to 40%. A combination of antiviral prophylaxis, social distancing, vaccination, and travel restriction reduced AR by more than 90% [23]. For a pandemic with Ro of 2.0, travel restriction in fact increased overall AR by 1% and peak AR by 20%. Household prophylaxis reduced AR by 35%, while vaccination reduced AR by 0 to 30%. A combination of antiviral prophylaxis, social distancing, vaccination, and travel restriction reduced AR by 80%.

### Disadvantages of individual measures

An individual-based stochastic model in Hong Kong looking at the effects of antiviral prophylaxis, case isolation and household quarantine reported that in a pandemic with Ro of 1.8 and AR of 74%, household quarantine could reduce AR to 49%; household quarantine and isolation to 43%; household quarantine with anti-viral prophylaxis to 44%; household quarantine, isolation and anti-viral prophylaxis to 40% which was recommended. Although adding contact tracing and quarantine of all contacts to the latter combination strategy reduced AR to 34%, the number of people under quarantine would be excessive. Therefore, contact tracing was not recommended [24].

Another study examining the effects of antiviral treatment and prophylaxis, home quarantine and social distancing based on a community of a million population with the assumption that pandemic influenza was introduced by an undetected airline passenger, found that if a pandemic Ro was 3.0, individual interventions would result in increased transmission while combination measures may break community transmission [25]. This was similarly shown by Ciofi and colleagues for a pandemic with Ro of 2.0 [23].

A deterministic compartmental model evaluating the effects of antiviral treatment and prophylaxis, vaccination, case isolation and air traffic reduction globally demonstrated that individual strategies such as case isolation and air travel restrictions may result in higher peak AR even though overall AR could be reduced [26].

A study in Taiwan evaluated the effects of enhanced ventilation, use of respiratory mask and vaccination on pandemic influenza transmission in a school [27]. Vaccination alone of 80% of children was effective in preventing the spread of the virus but this was only if a suitable vaccine was available, which is often not the situation. A combination of masks and ventilation, or a combination of vaccination and masks achieved similar effectiveness [27].

## Discussion

Many modeling studies were performed as a result of H5N1 influenza threat and an impending pandemic, but all have used parameters based on historical pandemics and existing studies on the influenza transmission. In addition, these studies provided sensitivity analyses across a wide range of influenza parameters. As such, they are directly relevant to the 2009 influenza pandemic which has an Ro of between 1.2 to 1.6 [28], similar to the 1957 and 1968 influenza pandemic [16], and for future pandemics. At the same time, the 2009 influenza pandemic provides the opportunity to study unknown variables to validate and refine these models.

All of these modeling studies in various settings, and using different models and assumptions, consistently show that combination strategies are more effective compared to individual strategies. Given the lack of good experimental, observation or controlled studies on these strategies, and the difficulties of performing trials during a pandemic, it is difficult for policy makers to know the effectiveness of their policies. These modeling studies provide policy makers with a suggestion of the effectiveness of different combination strategies. At the same time, new models will have to be developed using local data to provide realistic outcomes for local settings. The diverse methodology available from these studies provides sufficient information for countries to build and validate their results locally.

Although the use of individual-based and other stochastic models provide better data resolution, deterministic models mentioned in this review show similar outcomes [18,22,23,27]. These deterministic or simple stochastic compartmental models are much easier to build and may provide rapid results for policy making. This is especially true in countries where the vast amounts of data required for individual-based and complex stochastic models may not be available compared with high-income countries where most sophisticated models were built.

The use of combination strategies necessitates the availability of resources and feasibility for each individual component. For example, stockpiling of pharmaceutical agents is an integral part of preparedness plans and currently widely adopted in well-resourced countries. The increase in anti-viral drug resistance underscores the importance of combination drug use and provides policy makers with recommendations for their stockpiles [15]. Combination stockpiles of sufficient amounts of different antiviral drugs such as oseltamivir, zanamivir and adamantanes will allow for early combination chemotherapy or sequential multidrug therapy which was modeled to be effective against antiviral resistance when a small secondary stockpile was used to augment a primary stockpile [15]. The United States Federal stockpile is composed of 80% oseltamivir and 20% zanamivir, and several million doses of rimantadine from previous stockpiles [29]. The United Kingdom has purchased additional antiviral drugs to ensure it has a total stockpile for 50% of its population, comprising 68% oseltamivir and 32% zanamivir [30]. Bacterial pneumonia results in substantial morbidity and mortality among pandemic influenza cases [31,32]. Antibiotics should therefore be considered for stockpiling [31]. Stockpiles should take into account common locally circulating bacteria, and recommended amounts range from 10 to 25% of the population [33]. In contrast to antiviral drugs that are not widely used, antibiotics can be part of a rolling stockpile which ensures sufficient stockpiles without expiry issues. Vaccination against bacterial infections should likewise be considered.

From the effectiveness of combination strategies in reducing global spread of influenza or resistant viruses [12-15], resource-rich countries should consider redistributing their resources for the greater global benefit and their own benefit if they have yet to be affected by the pandemic. Controlling local outbreaks through combination strategies can reduce global spread, and countries affected early during the pandemic should be provided with assistance [13].

Vaccines are part of many combination strategies and modeling has shown that introduction of a vaccine four months after the pandemic virus has arrived has limited effectiveness, while stockpiling prototype pandemic vaccines could reduce overall AR [16]. Therefore countries were stockpiling H5N1 vaccines as candidate pandemic vaccines [34,35]. However, if the pandemic influenza virus is totally different from the vaccine virus, the vaccines would be of negligible effectiveness. Investments are needed to develop new vaccines with greater cross-protection against conserved viral regions; vaccine libraries to quickly produce candidate vaccines; better adjuvants and antigen-sparing strategies to increase production capacity; and modes of administration for improved immunogenicity and cross- protection [36,37].

Although some individual strategies may seem very effective, they may not be feasible and models assist policy makers in avoiding potentially disastrous decisions. Social distancing has been widely used in epidemics [7] but their impact remains unclear and highly dependent on disease severity, transmission, and risk groups affected. Local interventions such as school closures may be effective if done early, decisively, and for prolonged periods [20,38-40]. A United Kingdom model based on a 1957-like pandemic showed more than 20% case reduction if the Ro were low (<2) and schools were closed early, but less than 10% case reduction in pandemics with high Ro [38]. A French study showed that prolonged closure and limiting contact among children outside school may reduce cases by 17% and peak AR by 45% [39]. However, school closures and limiting social contact may be socio-economically difficult to achieve. Another study found that total closure of schools and workplaces reduced AR by 95%. However, the socio-economic impact would be unimaginable [20]. Similarly, most modeling studies found that travel restrictions alone did not impact overall AR [13,16,19,23]. Reducing air travel has been modeled to be effective in delaying pandemic spread if nearly 100% reduction can be achieved [13,16], and will be difficult if not impossible to achieve [41]. If used alone, local epidemic severity may increase because restriction-induced travel delays can push local outbreaks into high epidemic season [14].

Although combination strategies are more effective than individual measures, not all combination strategies may be feasible. Active surveillance, isolation of cases, and quarantine of close contacts are important interventions during epicenter containment. These interventions may reduce the Ro of the disease to below one and contain the outbreak. However, it is often difficult to ensure total compliance with these measures and if used alone, will result in missed cases due to surveillance failures, isolation facility exposures, and quarantine failures as shown in the SARS experience [42]. A Hong Kong modeling study found that although contact tracing and quarantine of all contacts was effective, it was not feasible because the number of people under quarantine would be excessive [24]. Therefore combination strategies enable policy makers to leverage on the effectiveness of some measures and reduce potential negative impact of others.

For combination strategies to work, they have to be tailored for each scenario at organizational, community, national, and international levels. To facilitate integration of interventions into effective combination strategies, more evidence is needed through targeted research, for example, the effectiveness of non-pharmaceutical interventions (e.g. personnel cohorting, school closures or reduction in air travel). In the absence of definitive studies, mathematical modeling studies provide an effective means of assessing the effectiveness of these strategies.

A limitation of this study is the restriction of our searches to the PubMed database. While we have made attempts to include additional articles from snowball searches, there is the potential for other published or unpublished studies to be missed from other databases and private sources. Other intrinsic limitations of modeling studies exist, and include the fact that they are based on theoretical epidemiology and not fully based on clinical or epidemiological evidence. For example, widespread use of pandemic vaccines raises safety concerns, and widespread use of antiviral drugs raises concern for antiviral resistance. Viral transmission during treatment with anti-viral drugs is also not well understood. It is therefore important to perform clinical and epidemiological studies during pandemic or seasonal influenza to understand the effectiveness and impact of these interventions. Models are also highly dependent on the assumptions and input variables, and are specific for a local context. However, if these limitations are understood by decision makers, modeling provides a reflection of the possible outcomes, helps to delineate possible strategies for inclusion, and avoids costly errors.

## Conclusion

Modeling studies show that combination strategies increase the effectiveness of individual strategies, guard against individual failures, and may reduce socio-economic impact. In the initial phases of an influenza pandemic, combination strategies provide the opportunity to contain the novel virus or delay its spread, allowing unaffected areas within a country and other countries to activate preventive strategies. During a pandemic, combination strategies allow for different strategies to have synergistic effect in reducing the impact of pandemic influenza, and the socio-economic impact of individual interventions. Finally, combination strategies protect against failure of individual interventions and should be considered in preparedness plans.

## Abbreviations

AR: Attack rate; RAR: Resistant attack rate; Ro: Reproductive number; US: United States; WHO: World Health Organization.

## Competing interests

VJL has received unrelated research support from GlaxoSmithKline. AWS has received honoraria and reimbursements to attend conferences by GlaxoSmithKline, Novartis, and Sanofi Pasteur.

## Authors' contributions

VJL contributed to the design, data collection, and manuscript writing. DCL and AWS contributed to the design and manuscript writing of this study.

## Appendix

### Description of key variables used in the models

Reproductive number (R) - Number of secondary infections generated by a single primary infection. The basic reproductive number (Ro) represents this number when the entire population is susceptible.

Anti-viral treatment - Treatment of individuals infected with influenza. Most of the studies use neuraminidase inhibitors such as oseltamivir as the drug of choice.

Anti-viral prophylaxis - Administration of anti-viral drugs to well contacts to prevent influenza infection. Prophylaxis here refers to post-exposure prophylaxis in a circumscribed area (household, school, workplace, geographical area).

Vaccination - Administration of an influenza vaccine to prevent influenza infections.

Quarantine - Segregation of well individuals exposed to influenza to prevent spread. Area quarantine is segregation of a geographical area with influenza cases within. Household quarantine is segregation of the household where a case has occurred.

Travel restrictions - Reduction in travel (air or border travel) by a quantum mentioned in the text.

Social distancing - Reduction in contact through strategies such as school and workplace closures, travel reductions, reduction in mass gatherings, behavioral changes in reducing contact, as mentioned in the text.

## Pre-publication history

The pre-publication history for this paper can be accessed here:

http://www.biomedcentral.com/1741-7015/7/76/prepub

## Supplementary Material

Additional file 1 **Table S1**. Combination strategy modeling studies to reduce the pandemic spread. Click here for file

Additional file 2 **Table S2**. Combination Strategy Modeling Studies to Mitigate the Pandemic Impact. Click here for file
