# Supplementary material for: Combination strategies for pandemic influenza response - a systematic review of mathematical modeling studies
Source: BMC Med. 2009 Dec 10;7:76. doi: 10.1186/1741-7015-7-76 (PMC2797001; doi:10.1186/1741-7015-7-76)
Supplement: Additional file 1 — Table S1. Combination strategy modeling studies to reduce the pandemic spread. [file 1741-7015-7-76-S1.DOC]

**Table S1: Combination strategy modeling studies to reduce the pandemic spread.**

| ***Authors / Source*** | ***Simulation Model Type*** | ***Strategy*** | ***Country, WHO Pandemic Alert Phase*** | ***Ro*** | ***Strategies Compared*** | ***Outcome Measures*** | ***Brief Results*** |
| --- | --- | --- | --- | --- | --- | --- | --- |
| **Longini et al, Science, 2005**  **9** | Stochastic individual-based model | Containment of pandemic influenza epicenter | Thailand, 4 | 1.1 to 2.4 | Household, school, workplace antiviral prophylaxis, pre-pandemic vaccination, area quarantine | 1. Cases 2. Containment proportion 3. Escapes | - Quarantine + pre-pandemic vaccination, + anti-viral prophylaxis effective in containing virus of up to Ro = 2.4 compared to ≤1.7 for individual strategies |
| **Ferguson et al, Nature, 2005**  **10** | Stochastic individual-based model | Containment of pandemic influenza epicenter | Thailand, 4 | 1.1 to 1.9 | Geographical antiviral prophylaxis, school and workplace closure for 21 days, area quarantine for 21 days | 1. Cases 2. Probability of elimination | - Blanket anti-viral prophylaxis of entire country will contain virus with Ro<3.6 but not feasible - Combined anti-viral prophyalxis + school and workplace closure + area quarantine is 92% effective (95% CI 91% to 97%) at containing virus with Ro = 1.9 and allows for greater surveillance errors, compared to Ro <1.25 to 1.7 for individual measures |
| **Colizza et al, PLOS Med, 2007 11** | Stochastic meta-population compartment model | Global cooperative strategies | Global, 4 onwards | 1.1 to 2.3 | Redistribution of anti-viral stockpiles | 1. Cases 2. Days to arrival 3. Days to peak | - In the event that anti-viral stockpiles are limited, non-cooperative strategies can only contain pandemics with Ro<1.5. - Cooperative strategies (where countries redistribute 25% of the drugs to other countries in need) can contain pandemics up to Ro = 1.9 and even at higher Ro = 2.3 reduces overall AR by 25%. |
| **Cooper et al, PLOS Med, 2006**  **12** | Stochastic meta-population compartment model | Air travel versus local measures | Global, 4 onwards | Not applicable | Air travel and local interventions (isolation, behavior change, antiviral use) to reduce influenza transmission | 1. Days to peak | - Even with 99.9% of air travel suspended, epidemics in individual countries would be delayed by 102 days (IQR 61, 133). - Reduction in transmission of influenza by 40% using combination of local strategies could delay the pandemic’s spread by 262 days (IQR 105, 349). |
| **Epstein et al, PLOS One, 2007**  **13** | Stochastic meta-population compartment model | Air travel and vaccination | United States, 4 onwards | 1.4 to 1.7 | Travel restrictions and vaccination | 1. Cases 2. Days to arrival 3. Days to peak | - When travel restrictions are imposed with R_0_ = 1.7, mean days to arrival of the pandemic increased by two to three weeks if originates in Hong Kong or Sydney but no impact if originates in London. - Vaccination-only does not substantially impact FPT but reduces total number of cases by 27 to 81% depending on country of origin. - Combination of vaccination and travel restrictions delays arrival by 0 to 5 weeks and reduces cases by 43 to 84% |
| **Wu et al, PLOS Med, 2009**  **14** | Stochastic individual-based multiple-compartment model | Anti-viral resistance | Global,  4 onwards | 1.8 | Treatment with oseltamivir, zanamivir, and adamantanes | 1. Overall attack rate (AR) 2. Resistant attack rate (RAR) | - At probability of emergence of drug resistance (pA) of 0.1, 40% treatment and Ro = 1.8, monotherapy has AR 72% (95% CI 71%, 73%) and RAR 66% (95% CI 60%, 71%), early combination chemotherapy has AR 63% (95% CI 63%, 63%) and RAR 18% (95% CI 18%, 18%), sequential multi-drug chemotherapy has AR 63% (95% CI 63%, 33%) and RAR 17% (95% CI 15%, 18%). |
