# Supplementary material for: Combination strategies for pandemic influenza response - a systematic review of mathematical modeling studies
Source: BMC Med. 2009 Dec 10;7:76. doi: 10.1186/1741-7015-7-76 (PMC2797001; doi:10.1186/1741-7015-7-76)
Supplement: Additional file 2 — Table S2. Combination Strategy Modeling Studies to Mitigate the Pandemic Impact. [file 1741-7015-7-76-S2.DOC]

**Table S2: Combination Strategy Modeling Studies to Mitigate the Pandemic Impact.**

| **Authors / Source** | **Simulation Model Type** | **Strategy** | **Country, WHO Pandemic Alert Phase** | **Ro** | **Strategies Compared** | **Outcome Measures** | **Brief Results** |
| --- | --- | --- | --- | --- | --- | --- | --- |
| Ferguson et al, Nature, 2006  15 | Stochastic individual-based model | Combination of pandemic strategies | United States and Great Britain,  5 & 6 | 1.4-2.0 | External or internal travel restrictions, school and workplace closures until 3 weeks after last detected case, antiviral treatment and household prophylaxis, household quarantine for 14 days | 1. Overall AR 2. Peak daily AR 3. Days to peak | - With Ro of between 1.7 to 2.0, external or internal travel restrictions alone delays spread by 3 to 4 weeks but only if 99% effective. - Combination treatment + household, school, work prophylaxis + school closure + effective border controls reduces overall AR by >70% and peak AR by >90%, compared to reduction in AR by <35% and park AR by <45% with individual strategies |
| Halloran et al, Proc Natl Acad Sci USA, 2008  16 | Stochastic individual-based model | Combination of pandemic strategies | Chicago, United States, 5 & 6 | 1.9-3.0 | Antiviral treatment and household prophylaxis, case isolation, quarantine of contacts for 10 days, school closure, workplace and community social distancing (closing theaters, reduced visits to restaurants, shops and public locations, banning mass gathering) | 1) Attack rate | - Social distancing alone reduced overall AR by 40-65% for Ro=3.0 to 60% for Ro=1.9 - Combination of treatment + prophylaxis + case isolation + contact quarantine + school closure + social distancing reduced overall AR of 53-85% for virus with Ro=3.0 and >90% for Ro=1.9 |
| Duerr et al, BMC Infect Dis, 2007  17 | Deterministic multiple compartment model | Combination of pandemic strategies | Germany, 5 & 6 | 2.5 | Case isolation, anti-viral treatment, social distancing (school and day care center closure, canceling mass gathering events, behavioral changes) | 1. Cases 2. Days to peak | - Case isolation + social distancing + anti-viral treatment delays peak by 1 month and reduces overall AR by 40%, compared to delay for <2 weeks and reduction in AR by 20% with individual strategy |
| Germann et al, Proc Natl Acad Sci USA, 2006  18 | Stochastic individual-based compartment model | Combination of pandemic strategies | United States, 5 & 6 | 1.6-2.4 | Antiviral prophylaxis (household, school, workplace), vaccination, continuous school closure, social distancing (travel restriction, quarantine, behavioral changes) | 1. Cases 2. Days to peak | - Travel restrictions are generally ineffective - For Ro=1.6, combination of all measures will reduce cases by almost 100% compared to 23% for social distancing to 99% for unlimited prophylaxis or best vaccination program - For Ro=2.4, combination of all measures will reduce cases by 99.8% compared to 6.3% for social distancing to 64% for unlimited prophylaxis and 34.2% for best vaccination program |
| Wu et al, PLOS Med, 2006  23 | Stochastic individual-based model | Household-based strategies | Hong Kong, 5 & 6 | 1.8 (range 1-3) | Contact tracing, case isolation, household antiviral prophylaxis, household quarantine until 7 days from last case | 1. Cases 2. Attack rate 3. Days to peak | - For Ro=1.8, combination of all measures reduce overall attack rate (AR) by 55% compared to 33% with quarantine only |
| Roberts et al, J R Soc Interface, 2007  24 | Stochastic meta-population compartment model | Combination of pandemic strategies | New Zealand, 5 & 6 | 1.1-3.0 | Targeted antiviral treatment and household prophylaxis (TATP), social distancing (school and workplace closure), household quarantine | 1. Attack rate 2. Reduction in reproductive number (R) | - For Ro=2.0, individual interventions would keep effective R between 0.95 to 2.0. Combination strategies would reduce R to between 0.4 to 0.8. |
| Nuno et al, J R Soc Interface, 2007  42 | Stochastic meta-population compartment model | Combination of pandemic strategies | US, UK, Netherlands, 5 & 6 | 1.6-2.4 | Transmission control measures (increased personal hygiene, isolation of infected individuals), antiviral treatment and prophylaxis, vaccine | 1. Cases 2. Hospitalizations 3. Deaths | - Transmission control measures alone reduces infections, hospitalizations and deaths by about 30% - Antiviral drugs and vaccines in sufficient quantities can lead to >99% decrease in outcomes - Combination of all 3 measures reduce impact by an additional 40-80% compared to antiviral drugs and vaccines only |
| Milne et al, PLOS One, 2008  20 | Stochastic individual-based model | Combination of non-pharmaceutical strategies | Albany, Australia, 5 & 6 | 1.5-2.5 | School closures, case isolation, workplace non-attendance, community contact reduction | 1. Attack rate 2. Peak daily AR | - Combination of all 4 strategies reduced the overall AR by >90%, compared to 15% to 40% with individual interventions |
| Flahault et al, Vaccine, 2006  25 | Deterministic compartment model | Combination of pandemic strategies | Global, 5 & 6 | 1.85-3.4 | Vaccination, case isolation, antiviral treatment and prophylaxis, reduction in air travel | 1. Attack rate | - Case isolation reduces AR by 9%, air travel restrictions by 1% - Addition of treatment to the above reduced AR by additional 10%, while addition of vaccination and treatment reduced AR by additional 60% |
| Carrat et al, BMC Med, 2006  19 | Stochastic individual-based multiple-compartment model | Combination of pandemic strategies | France, 5 & 6 | 2.07 | Vaccination, antiviral treatment and household prophylaxis, quarantine, school and workplace closures | 1. Attack rate 2. Days to peak | - Combination of anti-viral treatment, prophylaxis of household contacts, and household quarantine reduced AR by 83% (range 75% to 99%). - Treatment along reduced AR by 7% (range 5-9%), while household prophylaxis reduced AR by 23% (20-24%) - Only total closure of schools and workplaces was as effective, reducing AR by 79% (61-99%) |
| Ciofi et al, PLOS One, 2008  22 | Deterministic compartment model | Combination of pandemic strategies | Italy, 5 & 6 | 1.4-2 | Vaccination, household antiviral prophylaxis, air travel restrictions, closure of schools and workplaces (non-essential public offices) for 4 weeks starting from 4 weeks after pandemic | 1. Attack rate 2. Peak daily AR 3. Days to peak | - Ro=1.7, air travel restriction of 90% reduced AR by 0%; closure of schools/workplaces by 0%, prophylaxis by 49.9%, vaccination within 1 month by 42.2%. - Ro=2.0, air travel restriction of 90% reduced AR by 0%; closure of schools/workplaces by 0%, prophylaxis by 35.7%, vaccination within 1 month by 30.0%. - Combination of prophylaxis, school/workplace closures, vaccination, and air travel restrictions reduces AR by >90% for all Ro |
| Rizzo et al, Epidemiol Infect, 2008  21 | Deterministic compartment model | Combination of pandemic strategies | Italy, 5 & 6 | 1.8 (range 1.6-2,0) | Household antiviral prophylaxis, vaccination, social distancing (closure of schools for 3 weeks, public offices for 4 weeks, and public meeting places for 8 weeks) at 2, 4 and 8 weeks after pandemic | 1. Attack rate 2. Avoided cases | - For pandemic with 35% AR, vaccination reduced AR by 0.8 to 21.9%, prophylaxis by 0.3 to 5.4%, and social distancing by 0.3 to 1.7% - Combination of all 3 methods reduced AR by 5.0 to 33.6% |
| Chen and Liao, Epidemiol Infect, 2007  26 | Deterministic compartment model | Combination of strategies in schools | Taiwan, 5 & 6 | 2.8 – 16.9 across different childhood age groups | Wearing of masks, ventilation of schools, vaccination | 1) Cases  2) Peak of pandemic | - Masks and ventilation reduces AR by 25-35% and delays epidemic peak by 10-15 days. - Vaccination, combination of masks and ventilation, or combination of vaccination and masks prevented epidemics from occurring |
